# Snacking behaviors and early childhood caries severity among Myanmar preschool children: a cross-sectional study

**DOI:** 10.3389/froh.2026.1940409

**Published:** 2026-09-04

**Authors:** Phue Wai Han, Kaung Myat Thwin, Wa Than Lin, Natcha Tassanapong, Sachiko Takehara, Hiroshi Ogawa

**Affiliations:** 1 Division of Preventive Dentistry, Faculty of Dentistry, Graduate School of Medical and Dental Sciences, Niigata University, Japan; 2 Division of Preventive Dentistry, Faculty of Dentistry, Graduate School of Medicine, Dentistry and Health Sciences, Niigata University, Japan; 3 Division of Dental Public Health, Faculty of Dental Medicine, Universitas Airlangga, Indonesia; 4 Committee for Oral Health Education, Myanmar Dental Association, Myanmar; 5 Department of Community Dentistry, Faculty of Dentistry, Thammasat University, Pathumtani, Thailand

**Keywords:** dental caries, child, parents, snack, diet, feeding behavior, Myanmar

## Abstract

**Background:**

Early childhood caries (ECC) is highly prevalent among preschool children, particularly in Southeast Asian countries. Although dietary intake is a well-established risk factor, evidence on how specific snacking behaviors contribute to caries severity remains limited. This study examined the associations of food pocketing habit, main snack type, snack frequency, snack timing, and combined snacking patterns with ECC severity among Myanmar preschool children.

**Methods:**

A cross-sectional analysis was performed using baseline data from an ongoing cohort study of 815 children aged 4−5 years from eight preschools in Yangon between December 2024 and February 2025. Dental caries was assessed using the International Caries Detection and Assessment System II (ICDAS II), with the highest per-child ICDAS II score (0–6) used as a continuous outcome. Parents completed structured questionnaires on sociodemographic characteristics, parental socioeconomic factors, oral health behaviors, and snacking behaviors. Children were further classified according to snack frequency (≤2 or ≥3 times/day) and timing (fixed or irregular) into four combined snacking pattern groups. Linear regression analyses were performed using four sequential adjustment models.

**Results:**

Sweet snack consumption (86.4%) and irregular snack timing (80.2%) were highly prevalent. Food pocketing and main snack type were not significantly associated with caries severity in any model. Snack frequency and timing were each associated with ICDAS II scores across all models (fully adjusted: B = 0.3, 95%CI:0.1-0.6; and B = 0.4, 95%CI:0.2-0.7, respectively). Among combined snacking patterns, the ≥3 times/day and irregular timing group showed the most consistent association with higher caries severity across all models (fully adjusted: B = 0.8, 95%CI:0.4-1.1).

**Conclusion:**

Snack frequency, timing, and certain combined snacking patterns were associated with ECC severity. ECC prevention programs may consider addressing not only snack content but also the frequency and regularity of snacking.

## 1 Introduction

Early childhood caries (ECC) remains a major public health concern, affecting nearly half of preschool children worldwide (1, 2). Untreated caries can have serious consequences, including chronic pain, infection, and impaired quality of life (1, 3). As a multifactorial disease, ECC is influenced by a range of modifiable risk factors, including oral hygiene practices, fluoride exposure, and dietary behaviors (4–7). Among these, snacking behaviors are one of the important modifiable factors because they determine the frequency and duration of acid attacks on tooth surfaces, thereby disrupting the balance between demineralization and remineralization (7, 8). While dietary sugar intake is a well-established risk factor for ECC (7, 9), caries risk may also be influenced by broader snacking behaviors. These behaviors comprise multiple characteristics, including snack type, food pocketing habit, snack frequency, and snack timing. Although previous studies have provided evidence linking snacking behaviors to ECC, most have focused on individual aspects of snacking behaviors (10–13). Some studies have shown that snack frequency and snack timing have each been associated with ECC (12, 13); however, the association between combined snacking patterns, based on joint categories of snack frequency and timing, and ECC severity remains largely unexplored.

The burden of ECC is particularly high in Southeast Asian countries (14). In Myanmar, one of these countries, previous studies have reported ECC prevalence ranging from 83% to 87% among preschool children (15–17). However, these epidemiological studies assessing dental caries in Myanmar have relied primarily on the decayed, missing, and filled teeth (dmft) index (15–18), which records only cavitated lesions and may underestimate the true extent of disease. In contrast, the International Caries Detection and Assessment System II (ICDAS II) enables the detection of both non-cavitated and cavitated lesions, allowing caries severity to be evaluated across the full spectrum of disease and providing greater sensitivity for identifying early-stage lesions (19).

To our knowledge, no previous study in Myanmar has investigated the association between joint categories of snack frequency and timing and ECC severity using ICDAS II. Addressing this knowledge gap may provide a more comprehensive understanding of how specific snacking patterns are associated with caries progression and help inform targeted prevention strategies in countries, where the high prevalence of ECC underscores the need to consider modifiable behavioral risk factors. Therefore, this study aimed to examine the associations of food pocketing habit, snack type, snack frequency, snack timing, and combined snacking patterns with ECC severity, as measured by ICDAS II, among Myanmar preschool children. We hypothesized that children with frequent and irregular snacking patterns would have greater caries severity than those with less frequent and fixed-time snacking patterns.

## 2 Materials and methods

### 2.1 Study setting and participants

This study was a cross-sectional analysis of baseline data obtained from an ongoing prospective cohort study of preschool children in Yangon, Myanmar. The sampling frame covered all four administrative districts of Yangon City: Northern, Eastern, Western, and Southern. To ensure geographic representation, two preschools per district were selected by the Department of Social Welfare, Ministry of Social Welfare, Relief and Resettlement, using a census-based random sampling approach. The detailed study design, setting, participant recruitment, and sampling procedures have been described previously in our published study (20). Baseline data collected between December 2024 and February 2025 were used. Ethical approval for this study was obtained from the Ethics Committee of Niigata University, Japan (Approval No. 2024-0142) on November 13, 2024. Written informed consent was obtained from the parents or legal guardians of all participating children before enrollment. In addition, assent was obtained from each child prior to participation. The study was conducted in accordance with the principles of the Declaration of Helsinki and applicable local regulations. Children were eligible if they were 4-5 years of age, enrolled in one of the selected preschools, and had parental or guardian consent. Children were excluded if they had chronic medical conditions, systemic diseases, or were taking medications that could affect oral health. All eligible children were invited to participate regardless of ethnicity or religion.

**Figure 1:**
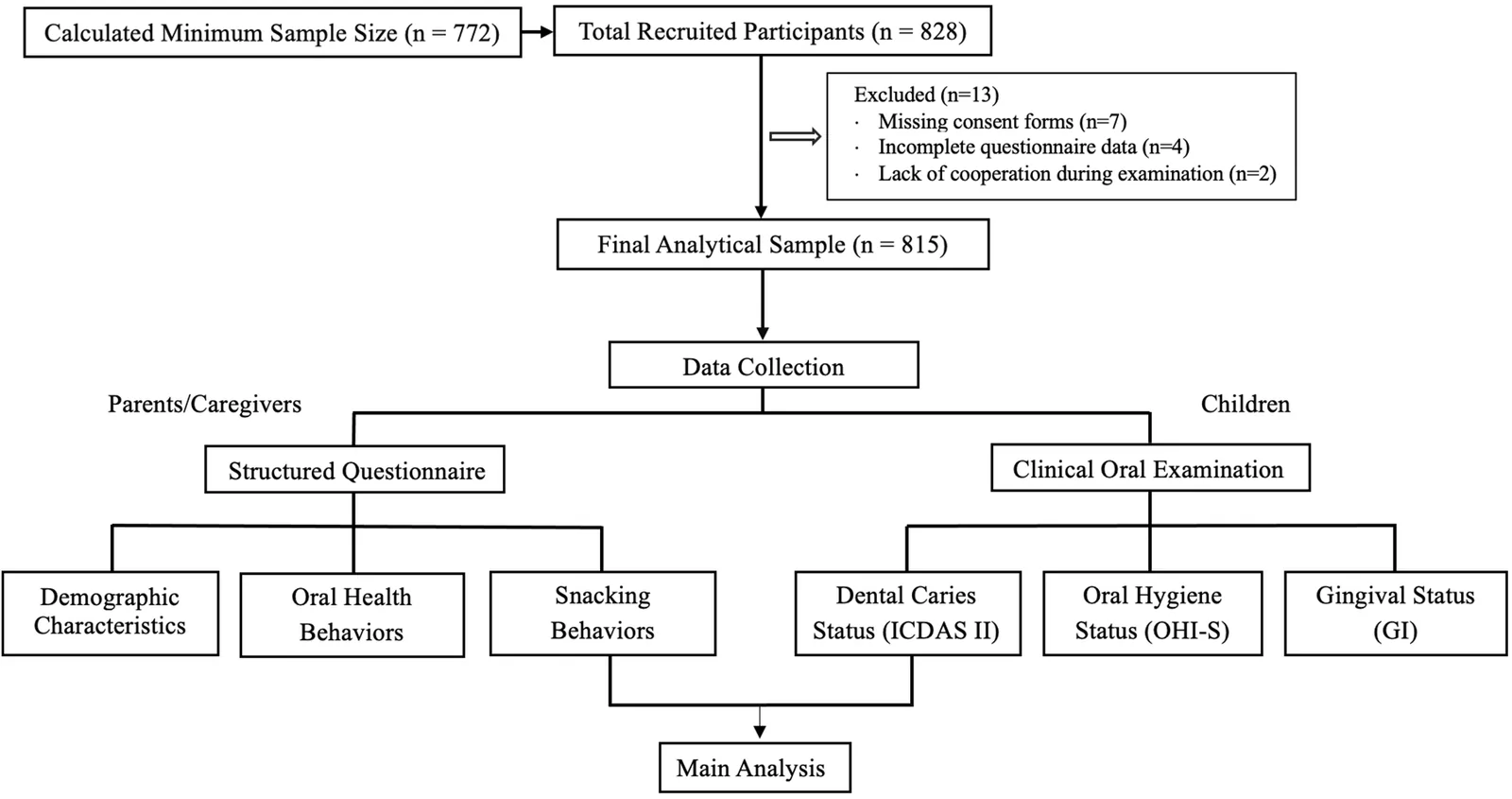
Flowchart of the study protocol.

The required sample size for the prospective cohort study was calculated using the formula for comparing two independent proportions, based on the expected proportions of caries increment reported in a previous study conducted in Myanmar (21). Assuming a significance level of 5%, a statistical power of 80%, and an anticipated dropout rate of 20%, the minimum required sample size was estimated to be 772 children. Of the 828 recruited children, 13 were excluded due to missing consent forms, incomplete questionnaire data or lack of cooperation during examination, leaving 815 parent-child pairs in the present analysis. Clinical oral examinations and structured questionnaires completed by parents or primary caregivers were conducted. An overview of the study protocol is presented in Figure 1.

### 2.2 Exposure variables

The primary exposure variables were the child's snacking behaviors, including food pocketing habit, main snack type, snack frequency, snack timing, and combined snacking patterns. Parents or primary caregivers completed a structured questionnaire that was distributed through preschool teachers. The completed questionnaires were returned to the teachers and collected by the research team. Any missing or incomplete responses were verified by returning the questionnaires to the respondents for completion. The questionnaire collected information on the child's demographic characteristics, parental socioeconomic factors, oral health behaviors, and dietary habits. Parents reported whether the child had a food pocketing habit (yes/no) and the types of snacks the child consumed. Main snack type was categorized as sweet (candies, chocolate, cookies, biscuits, and other sugar-containing snacks) or non-sweet (fruits and other snacks without added sugar) according to the snack most frequently consumed between meals.

Snack frequency was originally recorded as the usual number of snack occasions per day and was dichotomized into ≤2 times/day and ≥3 times/day for analysis based on previous evidence (8, 22). Snack timing was recorded as three groups according to the child's usual snacking schedule: fixed timing (snacks consumed at regularly scheduled times), non-fixed/irregular timing (snacks consumed at varying times without a regular schedule), and on demand (snacks consumed whenever requested by the child). For the further statistical analyses, the non-fixed/irregular timing and on-demand categories were combined into a single irregular timing group because both represented inconsistent snacking schedules. To examine snack frequency and timing jointly, participants were further classified into four combined snacking pattern groups: 1) ≤ 2 times/day and fixed timing (reference), 2) ≤ 2 times/day and irregular timing, 3) ≥ 3 times/day and fixed timing, and 4) ≥ 3 times/day and irregular timing.

### 2.3 Outcome variable

This study assessed dental caries severity through clinical oral examinations using the ICDAS II and the highest score obtained from each child was used to represent caries severity as the outcome variable. This approach was used to characterize each child's maximum caries severity rather than the cumulative disease burden, and consistent with a previously described approach (23). Examinations were conducted by four calibrated dentists with acceptable intra- and inter-examiner reliability following a standardized examination protocol (20).

All clinical examinations were performed under field conditions at the participating preschools with the knee-to-knee method, using disposable dental mirrors and WHO-CPI probes under natural daylight or handheld light. Tooth surfaces were cleaned and dried with sterile gauze and cotton rolls before caries assessment, as described previously (20).

### 2.4 Covariates

Potential confounding variables included the child's age and sex, body mass index (BMI), toothbrushing frequency, fluoride toothpaste use, mouth rinsing after meal, history of dental visits, and parental socioeconomic factors. The child's height and weight were obtained from preschool health records, and BMI was calculated as weight (kg) divided by height squared (m^2^). Toothbrushing frequency was assessed as had not yet started toothbrushing, once daily, twice daily, and three or more times daily, and dichotomized as ≤1 time/day and ≥2 times/day for statistical analyses. Fluoride toothpaste use was recorded as use, do not use, or do not know, and dichotomized as yes (use) and no (do not use/do not know). Mouth rinsing after meal was classified as always, sometimes, or never, and subsequently recoded as yes (always/sometimes) or no (never). History of dental visits included regular check-ups, visits for dental problems, and no previous dental visits. This variable was then recoded as yes (regular check-ups/visits for dental problems) or no (no previous dental visits). Parental socioeconomic variables included highest parental education and monthly household income. These variables were selected as complementary indicators of socioeconomic position because parental education and household income have previously been associated with ECC among preschool children (24). Highest parental education was derived from paternal and maternal education and categorized as ‘high school or above’ if at least one parent had attained this level; otherwise, it was categorized as ‘middle school or below’. Monthly household income was dichotomized as low (<350,000 MMK/month) or high (≥350,000 MMK/month), based on the Myanmar Household Welfare Survey 2023 (25).

### 2.5 Statistical analysis

All statistical analyses were performed using IBM SPSS Statistics for Windows, Version 27.0 (IBM Corp., Armonk, NY, USA). The distribution of continuous variables was assessed using the Kolmogorov–Smirnov test. Categorical variables were summarized as frequencies and percentages, whereas continuous variables were presented as medians and interquartile ranges (IQRs) because of their non-normal distribution.

Differences in child-level ICDAS II scores according to the exposure variables were examined using non-parametric tests. The Mann–Whitney U test was used for variables with two categories, whereas the Kruskal–Wallis test was applied for variables with more than two categories. Among the combined snacking pattern groups, *post-hoc* analyses were further performed using Bonferroni correction to account for multiple comparisons.

Linear regression analyses were conducted to examine the associations of individual snacking behaviors and combined snacking patterns with highest per-child ICDAS II scores. Four sequential models were constructed: Model 1 was unadjusted; Model 2 was adjusted for age, sex, and BMI as prespecified covariates; Model 3 was further adjusted for toothbrushing frequency, fluoride toothpaste use, mouth rinsing after meal, and history of dental visits to account for oral health-related behavioral confounders; and Model 4 was additionally adjusted for parental socioeconomic factors. Results are presented as unstandardized regression coefficients (B) with 95% confidence intervals (CIs). For the sensitivity analysis, the proportional-odds assumption for ordinal logistic regression was assessed using the test of parallel lines. Because this assumption was violated, multinomial logistic regression was performed instead as a sensitivity analysis after the highest per-child ICDAS II score was grouped according to the merged ICDAS classification into ICDAS 0 (sound), ICDAS 1-2 (initial-stage caries), ICDAS 3-4 (moderate-stage caries), and ICDAS 5-6 (extensive-stage caries). Sound was the reference outcome category. Each snacking exposure was examined in a separate model using the same covariate adjustments as the primary analysis. An OR greater than 1 indicated greater odds of being in the specified caries-severity category rather than the sound category. Results are presented as adjusted odds ratios (aORs) with 95% confidence intervals (CIs). A *p*-value <0.05 was considered statistically significant.

## 3 Results

Among the 815 participants, children aged 5 years accounted for 51.8%, and 54.0% were girls. The median ICDAS II score and BMI were 5.0 (IQR: 3.0, 6.0) and 13.9 (IQR: 12.5, 15.6), respectively. Sweet snack consumption was highly prevalent (86.4%), and 50.4% of children exhibited a food pocketing habit. Irregular snack timing, including non-fixed (48.8%) and on demand (31.4%), was reported, while 49.3% consumed snacks ≥3 times/day. The most common combined snacking pattern was ≥3 times/day and irregular timing (43.3%), followed by ≤2 times/day and irregular timing (37.0%) (Table 1). Children who consumed snacks ≥3 times/day had significantly higher median ICDAS II scores than those consuming snacks ≤2 times/day. Similarly, children with irregular snack timing had significantly higher median ICDAS II scores than those with fixed snack timing. Significant differences in ICDAS II scores were also observed among the four combined snacking pattern groups, whereas no significant differences were found according to food pocketing habit or main snack type (Supplementary Table S1).

**Table 1:** General characteristics of participants (*n* = 815).

Variable	*n* (%) or Median (IQR)
Age	
4 years	393 (48.2)
5 years	422 (51.8)
Sex	
Boys	375 (46.0)
Girls	440 (54.0)
**BMI**	**13.9 (12.5, 15.6)**
Main snack type	
Non-sweet snacks	111 (13.6)
Sweet snacks	704 (86.4)
Pocketing habit	
Yes	411 (50.4)
No	404 (49.6)
Snack frequency	
≤ 2 times/day	413 (50.7)
≥ 3 times/day	402 (49.3)
Snack timing	
Fixed	161 (19.8)
Non-fixed	398 (48.8)
On-demand	256 (31.4)
Snacking pattern groups	
≤2 times/day and Fixed timing	112 (13.7)
≤2 times/day and Irregular timing	301 (37.0)
≥3 times/day and Fixed timing	49 (6.0)
≥3 times/day and Irregular timing	353 (43.3)
Brushing frequency	
≥ 2 times/day	366 (44.9)
≤ 1 time/day	449 (55.1)
Fluoride toothpaste use	
Yes	617 (75.7)
No	198 (24.3)
Mouth rinsing after meal	
Yes	595 (73.0)
No	220 (27.0)
Dental visit	
Yes	199 (24.4)
No	616 (75.6)
Highest parental education	
High school and above	583 (71.5)
Middle school and lower	232 (28.5)
Household income	
High	358 (43.9)
Low	457 (56.1)
**ICDAS II score**	**5.0 (3.0, 6.0)**

Values are presented as *n* (%) for categorical variables and median (IQR) for continuous variables. Snacking pattern groups are based on the combination of snack frequency and timing. BMI, body mass index; ICDAS II, International Caries Detection and Assessment System II; IQR, interquartile range.

In linear regression analyses of individual snacking behaviors, food pocketing habit and main snack type were not significantly associated with caries severity in any model (Table 2). In contrast, snack frequency and snack timing were each associated with ICDAS II scores across all models. Compared with children who consumed snacks ≤2 times/day, those consuming snacks ≥3 times/day were significantly associated with higher ICDAS II scores in the unadjusted model and this association remained significant after full adjustment (B = 0.3, 95% CI: 0.1-0.6). Likewise, irregular snack timing was associated with higher ICDAS II scores in the unadjusted model and remained significant in the fully adjusted model (B = 0.4, 95% CI: 0.2-0.7). The multinomial logistic regression sensitivity analyses partially supported the primary findings. Irregular snack timing was associated with greater odds of extensive-stage caries rather than sound dentition across all four models. However, snack frequency was not significantly associated with any caries-severity category in the sensitivity analysis (Supplementary Table S2).

**Table 2:** Linear regression analysis of the association between individual snacking behaviors and caries severity.

Variable	Model 1		Model 2		Model 3		Model 4	
	B (95% CI)	*p*-value	B (95% CI)	*p*-value	B (95% CI)	*p*-value	B (95% CI)	*p*-value
Pocketing habit								
No	Reference		Reference		Reference		Reference	
Yes	−0.2 (−0.4, 0.0)	0.080	−0.2 (−0.4, 0.0)	0.084	−0.2 (−0.4, 0.0)	0.111	−0.2 (−0.4, 0.0)	0.102
Main snack type								
Non-sweet snacks	Reference		Reference		Reference		Reference	
Sweet snacks	0.0 (−0.4, 0.3)	0.886	0.0 (−0.3, 0.4)	0.895	0.1 (−0.3, 0.4)	0.651	0.1 (−0.2, 0.4)	0.567
Snack frequency								
≤ 2 times/day	Reference		Reference		Reference		Reference	
≥ 3 times/day	0.5 (0.2, 0.7)	**<0**.**001**	0.4 (0.1, 0.6)	**0**.**003**	0.3 (0.1, 0.6)	**0**.**008**	0.3 (0.1, 0.6)	**0**.**010**
Snack timing								
Fixed	Reference		Reference		Reference		Reference	
Irregular	0.5 (0.3, 0.8)	**<0**.**001**	0.4 (0.1, 0.7)	**0**.**005**	0.4 (0.1, 0.7)	**0**.**004**	0.4 (0.2, 0.7)	**0**.**003**

Bold values indicate statistical significance *p* < 0.05. Model 1, Unadjusted; Model 2, Adjusted for age, sex, and BMI; Model 3, Additionally adjusted for brushing frequency, fluoride toothpaste use, mouth rinsing after meal and dental visits; Model 4, Additionally adjusted for highest parental education and monthly household income. Caries severity, assessed using the International Caries Detection and Assessment System II (ICDAS II), was used as the dependent variable.

When combined snacking patterns were examined, children with ≥3 times/day and irregular timing group demonstrated the most consistent association with higher ICDAS II scores across all regression models (Table 3). Notably, the ≤2 times/day and irregular timing group became significantly associated with higher ICDAS II scores after adjustment for potential confounders (Model 2: B = 0.4, 95% CI: 0.1-0.8; Model 3: B = 0.5, 95% CI: 0.1-0.8; Model 4: B = 0.5, 95% CI: 0.1-0.8), whereas no significant association was observed in the crude analysis. In contrast, although children in the ≥3 times/day and fixed timing group showed a positive estimate after adjustment, the association did not reach statistical significance. Sensitivity analyses showed a similar overall pattern of findings, both the ≤2 times/day and irregular-timing group and the ≥3 times/day and irregular-timing group had greater odds of extensive-stage caries rather than sound dentition across all four models, compared with the ≤2 times/day and fixed-timing group (Supplementary Table S3).

**Table 3:** Linear regression analysis of the association between combined snacking patterns and caries severity.

Variable	Model 1		Model 2		Model 3		Model 4	
	B (95% CI)	*p*-value	B (95% CI)	*p*-value	B (95% CI)	*p*-value	B (95% CI)	*p*-value
≤2 times/day and Fixed timing	Reference		Reference		Reference		Reference	
≤2 times/day and Irregular timing	−0.1 (−0.4, 0.1)	0.221	0.4 (0.1, 0.8)	**0**.**015**	0.5 (0.1, 0.8)	**0**.**009**	0.5 (0.1, 0.8)	**0**.**009**
≥3 times/day and Fixed timing	−0.1 (−0.6, 0.4)	0.607	0.4 (−0.1, 1.0)	0.145	0.5 (−0.1, 1.0)	0.102	0.5 (−0.1, 1.0)	0.109
≥3 times/day and Irregular timing	0.5 (0.3, 0.7)	**<0**.**001**	0.8 (0.4, 1.1)	**<0**.**001**	0.8 (0.4, 1.1)	**<0**.**001**	0.8 (0.4, 1.1)	**<0**.**001**

Bold values indicate statistical significance *p* < 0.05. Model 1, Unadjusted; Model 2, Adjusted for age, sex, and BMI; Model 3, Additionally adjusted for brushing frequency, fluoride toothpaste use, mouth rinsing after meal and dental visits; Model 4, Additionally adjusted for highest parental education and monthly household income. Caries severity, assessed using the International Caries Detection and Assessment System II (ICDAS II), was used as the dependent variable.

## 4 Discussion

This cross-sectional study examined the associations between snacking behaviors and the severity of ECC among preschool children in Myanmar using the ICDAS II scoring system. The key findings revealed that snack frequency and snack timing were each associated with higher ICDAS II scores after adjustment for the specified covariates, whereas food pocketing and main snack type were not. Among the combined snacking patterns, the ≥3 times/day and irregular-timing group had the largest estimated difference compared with the ≤2 times/day and fixed-timing group, highlighting the potential importance of frequent, unscheduled snacking behaviors in relation to ECC. In this study, the median ICDAS II score of 5 indicates that many children already had at least one advanced cavitated lesion, highlighting the considerable severity of ECC in this population; however, this measure does not reflect the overall extent or total number of carious lesions per child. Although previous studies in Myanmar have reported ECC prevalence rather than lesion severity (15–17), both sets of findings consistently indicate a substantial burden of ECC among preschool children. Moreover, a high prevalence of cariogenic snacking behaviors was observed, with approximately half of the children consumed snacks ≥3 times/day, 80.2% had irregular snack timing, and 43.3% exhibited the combined pattern of frequent and irregular snacking. The high consumption of sweet snacks observed in this study may reflect the widespread availability and early consumption of sugary snack foods in Southeast Asia, where frequent sweet food intake has been recognized as a well-established ECC risk factor (5, 14, 26–28).

After adjustment for potential confounders (Model 4), higher snack frequency and irregular snack timing were each associated with greater caries severity, consistent with previous studies (12, 13). These suggest that the timing of snack consumption may be relevant to ECC beyond snack frequency alone. Among combined snacking patterns, the ≥3 times/day and irregular timing group showed the most consistent association with higher caries severity across all models, which is biologically plausible because frequent and irregular exposure to fermentable carbohydrates may increase acid challenges and limit opportunities for salivary clearance, buffering, and enamel remineralization between eating intervals (11, 29–33). The ≤2 times/day and irregular timing group was not significant in the crude model, the association became significant after adjustment for potential confounders, indicating that even ≤2 times/day frequency can elevate caries risk when snacks are consumed at irregular times. Conversely, the ≥3 times/day and fixed timing group showed a similar effect estimate but did not reach statistical significance, possibly because of the relatively small subgroup size (*n* = 49). Overall, these results suggest that irregular snack timing may be an important behavioral factor associated with more severe caries experience, regardless of snack frequency.

In contrast, no significant associations were observed between main snack type, food pocketing, and caries severity. This may be because caries development is influenced not only by snack type but also by the frequency, timing, and pattern of consumption, which may exert a greater effect on cariogenic challenge than the predominant snack type alone (5, 9, 34–36). Similarly, although food retention in the mouth may theoretically prolong acid production by increasing substrate contact with tooth surfaces (37), the dichotomous assessment of food pocketing (yes/no) may not have adequately captured its frequency or duration, which could be more relevant to caries risk. Additionally, caregiver-reported measures may be subject to recall error or misclassification, potentially attenuating true associations.

Taken together, these findings suggest that snack timing may be a relevant behavioral factor associated with ECC severity and should be considered alongside snack frequency. These findings are consistent with previous research highlighting the importance of snacking behaviors in the development of ECC and extend the existing evidence by considering both snack frequency and snack timing. Previous longitudinal evidence from Japan indicated that consuming between-meal snacks more than four times per day was associated with increased odds of dental caries among young children (12). Similarly, Zahid et al. (2020) reported that frequent consumption of sweet and processed snacks was associated with severe dental caries among Nepalese children (10). Building on these observations, the present study suggests that considering both snack frequency and snack timing may provide a more comprehensive assessment of behavioral risk for ECC than either factor alone.

This study has several strengths. Data included a relatively large sample of preschool children in Yangon and used standardized clinical examinations performed by calibrated dentists. Dental caries was assessed using the ICDAS II system, enabling evaluation across the full spectrum of lesion severity rather than relying solely on cavitated lesions. Lastly, to our knowledge, this is the first study in Myanmar to investigate the association of combined snacking patterns with ECC severity, providing novel evidence for behavioral risk assessment. Several limitations should also be acknowledged. First, this study employed a cross-sectional design, which causal relationships cannot be inferred. Longitudinal follow-up studies are needed to confirm the observed associations and clarify whether snacking behaviors contribute to the development of dental caries over time. Second, the reliance on parent-reported snacking behaviors introduces potential recall and social desirability biases, particularly for variables such as snack timing and food pocketing. Third, approximately one-third of children scored at the maximum ICDAS II (score 6), which compressed the variability of the outcome and may have underestimated the true associations among children with the most severe caries. Fourth, participants were recruited from preschools in a single city, Yangon, which may limit the generalizability of the findings. Furthermore, the potential clustering of children within preschools was not accounted for in the analyses, as a result, standard errors may be underestimated and confidence intervals overly narrow. Finally, snacking behaviors were assessed using relatively broad questionnaire categories, and detailed information on total sugar intake, snack portion size, sweetened beverage consumption, and snack timing relative to bedtime was not collected.

Despite these limitations, the present study may have important implications for ECC prevention programs in Myanmar and similar low-resource settings. While current strategies primarily emphasize reducing sugar intake and promoting fluoride use, our findings suggest that optimizing snack frequency and regularity may be equally important. Furthermore, identifying children who exhibit both high-frequency and irregular snacking patterns could help clinicians and public health practitioners facilitate the delivery of tailored dietary counseling and preventive oral health education. To this end, these findings may inform guidance encouraging caregivers to provide snacks at planned times and avoid frequent grazing between meals, as well as school-based oral health education programs in Myanmar promoting structured snack schedules through existing preschool mealtime routines.

## 5 Conclusion

Snack frequency, snack timing, and certain combined snacking patterns were associated with caries severity among Myanmar preschool children. Children with frequent and irregular snacking patterns showed the strongest association among those with other snacking patterns. These findings highlight the potential importance of considering both the frequency and regularity of snacking when developing strategies for the prevention of early childhood caries.

## Data Availability

The raw data supporting the conclusions of this article will be made available by the authors, without undue reservation.
