# Supplementary material for: Snacking behaviors and early childhood caries severity among Myanmar preschool children: a cross-sectional study
Source: Front Oral Health. 2026 Sep 4;7:1940409. doi: 10.3389/froh.2026.1940409 (PMC13592035; doi:10.3389/froh.2026.1940409)
Supplement: Supplementary file 1 [file Table1.docx]

**Supplementary Table 1. Binary association between snacking behaviors and caries severity**

| **Variable** | **ICDAS score** | |
| --- | --- | --- |
|  | **Median (IQR)** | ***p*-value** |
| Pocketing habit |  |  |
| No | 5.0 (3.0, 6.0) | 0.124 |
| Yes | 4.0 (3.0, 6.0) |  |
| Main snack type |  |  |
| Non-sweet snacks | 5.0 (3.0, 6.0) | 0.547 |
| Sweet snacks | 5.0 (3.0, 6.0) |  |
| Snack frequency |  |  |
| ≤2 times/day | 4.0 (3.0, 6.0) | **<0.001** |
| ≥3 times/day | 5.0 (3.0, 6.0) |  |
| Snack timing |  |  |
| Fixed | 4.0 (3.0, 6.0) | **<0.001** |
| Irregular | 5.0 (3.0, 6.0) |  |
| Snacking pattern groups |  |  |
| ≤2 times/day and Fixed timing | 4.0 (3.0, 5.8) | **0.005** |
| ≤2 times/day and Irregular timing | 4.0 (3.0, 6.0) |  |
| ≥3 times/day and Fixed timing | 4.0 (3.0, 6.0) |  |
| ≥3 times/day and Irregular timing | 5.0 (3.0, 6.0) |  |
| Adjusted *p*-value* | **0.021^a^**, 0.106^b^, **< 0.001^c^**, 0.891^d^, **0.002^e^**, 0.141^f^ | |

Note: Bold values indicate statistical significance *p*<0.05.

Analyses were performed using the Mann-Whitney U test or Kruskal-Wallis test.

*For the four snacking pattern groups, further analysis was performed by using Bonferroni-adjusted post hoc pairwise comparisons.

^a^ ≤2 times and Fixed vs ≤2 times and Irregular; ^b^ ≤2 times and Fixed vs ≥3 times and Fixed; ^c^ ≤2 times and Fixed vs ≥3 times and Irregular; ^d^ ≤2 times and Irregular vs ≥3 times and Fixed; ^e^ ≤2 times and Irregular vs ≥3 times and Irregular; ^f^ ≥3 times and Fixed vs ≥3 times and Irregular.

**Supplementary Table 2. Multinomial logistic regression analysis of the association between individual snacking behaviors and caries severity (Sensitivity analysis)**

| **Exposure Variables** | **Outcome Variables**  **(Ref: ICDAS 0)** | **Model 1** | | **Model 2** | | **Model 3** | | **Model 4** | |
| --- | --- | --- | --- | --- | --- | --- | --- | --- | --- |
|  |  | **OR (95% CI)** | ***p*-value** | **aOR (95% CI)** | ***p*-value** | **aOR (95% CI)** | ***p*-value** | **aOR (95% CI)** | ***p*-value** |
| Pocketing habit  (Ref: No) | ICDAS (1-2) | 1.7 (0.7, 4.0) | 0.241 | 1.7 (0.7, 4.2) | 0.229 | 1.7 (0.7, 4.2) | 0.250 | 1.7 (0.7, 4.3) | 0.245 |
|  | ICDAS (3-4) | 0.8 (0.4, 1.7) | 0.608 | 0.9 (0.4, 1.8) | 0.673 | 0.9 (0.4, 2.0) | 0.778 | 0.9 (0.4, 2.0) | 0.833 |
|  | ICDAS (5-6) | 0.8 (0.4, 1.6) | 0.444 | 0.8 (0.4, 1.6) | 0.492 | 0.8 (0.4, 1.7) | 0.560 | 0.9 (0.4, 1.7) | 0.577 |
| Main snack type  (Ref: Non-sweet snacks) | ICDAS (1-2) | 1.6 (0.5, 4.8) | 0.451 | 1.9 (0.6, 6.1) | 0.301 | 2.0 (0.6, 6.7) | 0.267 | 2.1 (0.6, 7.3) | 0.234 |
|  | ICDAS (3-4) | 1.7 (0.7, 4.4) | 0.286 | 2.0 (0.7, 5.4) | 0.173 | 2.3 (0.8, 6.5) | 0.102 | 2.4 (0.9, 6.8) | 0.101 |
|  | ICDAS (5-6) | 1.5 (0.6, 3.7) | 0.419 | 1.8 (0.7, 4.7) | 0.238 | 2.3 (0.8, 6.2) | 0.110 | 2.4 (0.9, 6.8) | 0.087 |
| Snack frequency  (Ref: ≤2 times/day) | ICDAS (1-2) | 0.5 (0.2, 1.2) | 0.101 | 0.4 (0.2, 1.0) | 0.060 | 0.5 (0.2, 1.2) | 0.110 | 0.5 (0.2, 1.3) | 0.138 |
|  | ICDAS (3-4) | 1.3 (0.6, 2.7) | 0.518 | 1.0 (0.4, 2.1) | 0.914 | 1.2 (0.5, 2.6) | 0.747 | 1.3 (0.6, 3.0) | 0.550 |
|  | ICDAS (5-6) | 1.7 (0.8, 3.5) | 0.172 | 1.3 (0.6, 2.8) | 0.570 | 1.4 (0.6, 3.2) | 0.423 | 1.5 (0.7, 3.5) | 0.327 |
| Snack timing  (Ref: Fixed) | ICDAS (1-2) | 1.9 (0.8, 4.6) | 0.181 | 2.5 (1.0, 6.6) | 0.057 | 2.5 (1.0, 6.6) | 0.064 | 2.5 (0.9, 6.8) | 0.078 |
|  | ICDAS (3-4) | 2.2 (1.0, 4.7) | **0.046** | 2.2 (1.0, 4.9) | 0.063 | 2.1 (0.9, 4.9) | 0.072 | 1.9 (0.8, 4.4) | 0.152 |
|  | ICDAS (5-6) | 3.4 (1.6, 7.4) | **0.002** | 3.2 (1.4, 7.2) | **0.005** | 3.2 (1.4, 7.4) | **0.005** | 3.0 (1.3, 7.0) | **0.011** |

Note: Caries severity was categorized according to the highest per-child ICDAS II score as ICDAS 0 (sound), ICDAS 1-2 (initial-stage caries), ICDAS 3-4 (moderate-stage caries), and ICDAS 5-6 (extensive-stage caries). Sound was the reference outcome category. ORs greater than 1 indicate greater odds of being in the specified caries-severity category rather than the sound category. Each snacking exposure was entered in a separate multinomial logistic regression model. Model 1, unadjusted; Model 2, adjusted for age, sex, and BMI; Model 3, additionally adjusted for toothbrushing frequency, fluoride toothpaste use, mouth rinsing after meal, and history of dental visits; Model 4, additionally adjusted for highest parental education and monthly household income. Results are presented as adjusted odds ratios (aORs) with 95% confidence intervals (CIs). Bold values indicate statistical significance at *p*<0.05. BMI, body mass index; ICDAS II, International Caries Detection and Assessment System II. The multinomial estimates should be interpreted cautiously because the sound reference group was small, resulting in imprecise estimates and relatively wide confidence intervals.

**Supplementary Table 3. Multinomial logistic regression analysis of the association between combined snacking patterns and caries severity (Sensitivity analysis)**

| **Exposure Variables** | **Outcome Variables**  **(Ref: ICDAS 0)** | **Model 1** | | **Model 2** | | **Model 3** | | **Model 4** | |
| --- | --- | --- | --- | --- | --- | --- | --- | --- | --- |
|  |  | **OR (95% CI)** | ***p*-value** | **aOR (95% CI)** | ***p*-value** | **aOR (95% CI)** | ***p*-value** | **aOR (95% CI)** | ***p*-value** |
| Ref: ≤2 times/day and Fixed timing | | | | | | | | | |
| ≤2 times/day and Irregular timing | ICDAS (1-2) | 2.3 (0.8, 7.0) | 0.133 | 2.5 (0.8, 7.6) | 0.110 | 2.3 (0.7, 7.3) | 0.154 | 2.2 (0.7, 7.3) | 0.182 |
|  | ICDAS (3-4) | 2.3 (0.9, 6.1) | 0.099 | 2.2 (0.8, 5.8) | 0.133 | 1.9 (0.7, 5.4) | 0.206 | 1.6 (0.6, 4.7) | 0.360 |
|  | ICDAS (5-6) | 3.7 (1.4, 9.9) | **0.010** | 3.5 (1.3, 9.4) | **0.015** | 3.3 (1.2, 9.2) | **0.022** | 3.0 (1.0, 8.5) | **0.042** |
| ≥3 times/day and Fixed | ICDAS (1-2) | 0.4 (0.1, 2.9) | 0.362 | 0.4 (0.1, 2.6) | 0.299 | 0.3 (0.0, 2.4) | 0.276 | 0.3 (0.1, 2.5) | 0.290 |
|  | ICDAS (3-4) | 1.3 (0.3, 5.1) | 0.756 | 1.0 (0.2, 4.1) | 0.987 | 1.0 (0.2, 4.2) | 0.978 | 1.1 (0.2, 4.6) | 0.938 |
|  | ICDAS (5-6) | 1.8 (0.4, 7.3) | 0.411 | 1.5 (0.4, 6.3) | 0.565 | 1.6 (0.4, 6.7) | 0.538 | 1.7 (0.4, 7.1) | 0.498 |
| ≥3 times/day and Irregular | ICDAS (1-2) | 0.9 (0.3, 2.8) | 0.858 | 1.0 (0.3, 3.1) | 0.939 | 1.0 (0.3, 3.4) | 0.986 | 1.0 (0.3, 3.5) | 0.954 |
|  | ICDAS (3-4) | 2.4 (0.9, 6.2) | 0.079 | 1.8 (0.7, 4.9) | 0.240 | 2.1 (0.7, 5.8) | 0.163 | 2.0 (0.7, 5.6) | 0.181 |
|  | ICDAS (5-6) | 4.6 (1.7, 11.9) | **0.002** | 3.7 (1.4, 9.9) | **0.009** | 4.0 (1.5, 11.2) | **0.007** | 4.0 (1.5, 11.1) | **0.008** |

Note: Caries severity was categorized according to the highest per-child ICDAS II score as ICDAS 0 (sound), ICDAS 1-2 (initial-stage caries), ICDAS 3-4 (moderate-stage caries), and ICDAS 5-6 (extensive-stage caries). Sound was the reference outcome category. ORs greater than 1 indicate greater odds of being in the specified caries-severity category rather than the sound category. Each snacking exposure was entered in a separate multinomial logistic regression model. Model 1, unadjusted; Model 2, adjusted for age, sex, and BMI; Model 3, additionally adjusted for toothbrushing frequency, fluoride toothpaste use, mouth rinsing after meal, and history of dental visits; Model 4, additionally adjusted for highest parental education and monthly household income. Results are presented as adjusted odds ratios (aORs) with 95% confidence intervals (CIs). Bold values indicate statistical significance at *p*<0.05. BMI, body mass index; ICDAS II, International Caries Detection and Assessment System II. The multinomial estimates should be interpreted cautiously because the sound reference group was small, resulting in imprecise estimates and relatively wide confidence intervals.
